# Direct observations of bedform migration driven by turbidity currents in a lacustrine channel

**DOI:** 10.1038/s41598-025-21833-6

**Published:** 2025-10-30

**Authors:** Gaétan Sauter, Damien Bouffard, Stefano C. Fabbri, Koen Blanckaert, Flavio S. Anselmetti, Katrina Kremer

**Affiliations:** 1 Institute of Geological Sciences, University of Bern, Oeschger Centre for Climate Change Research, Baltzerstrasse 1+3, CH-3012 Bern, Switzerland; 2 Department of Surface Waters - Research and Management, Swiss Federal Institute of Aquatic Science and Technology, 6047 Kastanienbaum, Switzerland; 3 Faculty of Geosciences and Environment, Institute of Earth Surface Dynamics, University of Lausanne, 1015 Lausanne, Switzerland; 4 Federal Office of Topography Swisstopo, Seftigenstrasse 264, 3084 Wabern, Switzerland; 5 TU WIEN Research Unit Hydraulic Engineering and Environmental Hydromechanics, Vienna, Austria; 6 Swiss Seismological Service, ETH Zurich, Sonneggstrasse 5, CH-8092 Zurich, Switzerland

**Keywords:** Hydrology, Limnology, Physical oceanography

## Abstract

Turbidity currents, gravity-driven sediment-laden flows, govern material transport and shape underwater landscapes across diverse environments. While extensively studied in marine settings, their dynamics in freshwater systems remain underexplored. We present multi-temporal, multi-scale observations from the Aare Delta of Lake Brienz (Switzerland), combining Acoustic Doppler Current Profiler measurements with high-resolution repeated bathymetric surveys to capture turbidity currents and resulting morphological changes. A single turbidity current reached near-bed velocities of 0.65 m/s and induced upslope migration of a cyclic step by at least 4 m within minutes. Characterisation of overall bedform migration across the channel network over five years highlights the cumulative imprint of turbidity currents and frames these short-term changes within the broader context of channel reworking. Together, our results show striking similarities in overall flow structure, bedform morphologies, and dynamics between freshwater and marine systems, underscoring the value of lacustrine settings as accessible, scaled-down environments for advancing turbidity current research.

**Supplementary Information:**

The online version contains supplementary material available at 10.1038/s41598-025-21833-6.

## Introduction

Turbidity currents are gravity-driven sediment-laden flows that move downslope due to their excess density compared to the surrounding water^1–4^. They play a key role in shaping underwater landscapes and driving geochemical cycles within lakes and oceans by redistributing sediments, nutrients, organic carbon, and oxygen from shallow to deep environments^5–8^. Given their substantial velocities and associated energy^4^, they have also been shown to damage underwater infrastructure such as fibre-optic cables and pipelines^9,10^. Their ecological and economic implications have spurred extensive scientific investigation into their dynamics^4^, despite the challenges of monitoring these flows and linking short-term events to long-term sedimentary processes.

Turbidity currents were first described in the late 19th century by Forel^11^, who hypothesised that the cold, turbid Rhône River, disappearing beneath the surface upon entering Lake Geneva (Switzerland/France), generated underwater currents. He suggested that these flows carved the deep canyon already known from early bathymetric surveys, a theory confirmed decades later^12^. Since then, research has focused primarily on oceanic settings, while freshwater systems have received comparatively little attention^4^. Yet lakes offer notable advantages, as their shallower depths and accessibility make field operations easier and less costly, while fewer external forcing factors provide tighter constraints on potential triggering mechanisms. Freshwater systems may therefore serve as natural-scale laboratories bridging the gap between controlled flume experiments and monitoring of large marine environments^13^.

Although the mechanisms that generate turbidity currents are consistent across a variety of environments^14^, their characteristics might differ between marine and lacustrine settings. One important distinction lies in scale: lacustrine systems typically span only a few km^13,15–17^, whereas oceanic systems can extend tens to hundreds of km^1,18^. Triggers also differ^19,20^: processes such as storm waves^21–24^ and tides^19,25^ have been shown to initiate turbidity currents in the oceans, whereas these drivers play a more limited role in lakes. In the ocean, riverine freshwater encounters denser saline water, creating strong density contrasts^26^. In lakes, however, inflows and ambient water usually have similar densities^11,12,26,27^. When river water is colder and/or sediment-laden, it plunges along the lakebed, a process known as hyperpycnal plunging^28^. This can occur at relatively low sediment concentrations and is therefore considered a primary trigger of turbidity currents in lacustrine environments^26^. Once initiated, physical and chemical differences in ambient water properties (saline versus freshwater) may shape how flows evolve^29^ by affecting turbulence, entrainment, and mixing rates^30^. In saline environments, enhanced flocculation of fine particles can also modify settling rates^31,32^. Understanding these differences is important when comparing observations between lacustrine and marine settings.

**Fig. 1:**
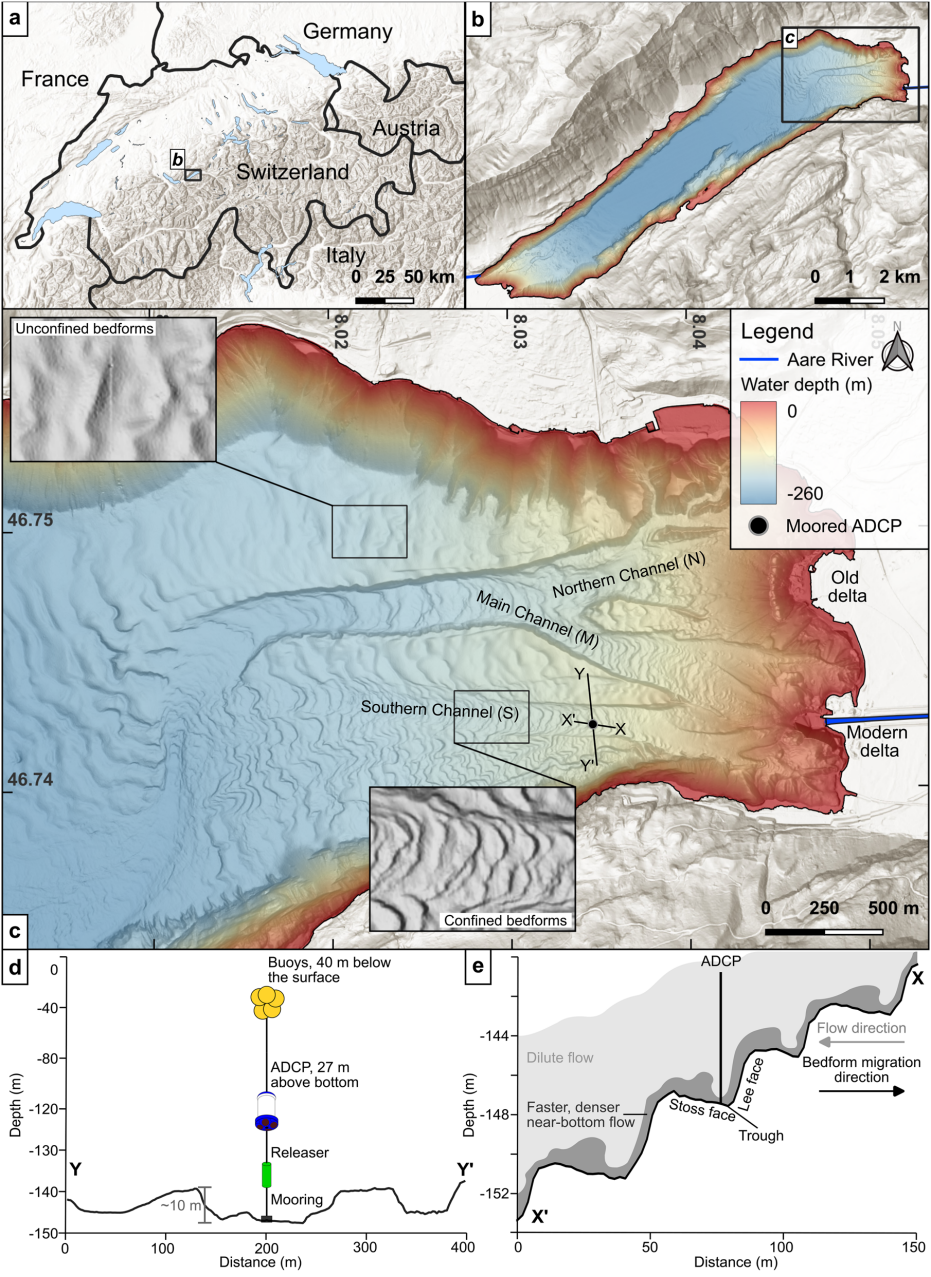
(**a**) Location of the study site in Switzerland. The black rectangle outlines the extent shown in (**b**). (**b**) High-resolution bathymetric map of Lake Brienz acquired in 2018 (2-m grid size), with multidirectional shaded relief superimposed on the slope map. Colours represent water depths (m). A black rectangle outlines the area detailed in (**c**). (**c**) Bathymetric map of the proximal Aare delta, showing the Aare River inflow and the locations of the moored ADCPs. Cross-channel (Y-Y’) and thalweg (X-X’) profiles are presented in (**d**) and (**e**), respectively, are indicated. (**d**) Cross-channel profile (Y-Y’) showing the ADCP setup used in this study and the Channel S height at this location. (**e**) Channel thalweg profile (X–X’) with a schematic turbidity current. Maps generated in QGIS 3.34 (https://qgis.org) and edited in Inkscape 1.4 (https://inkscape.org). Sources: Federal Office of Topography Swisstopo and Esri World Hillshade via QuickMapServices. CRS: EPSG:4326.

To assess whether lacustrine turbidity currents are relevant analogues for oceanic systems, further research in freshwater systems is needed. Our study addresses this knowledge gap by examining the Aare Delta (Figs. 1a-c) in Lake Brienz (Switzerland) ^33–36^. We combine high-resolution repetitive multibeam bathymetry with flow measurements (Figs. 1c, d) from Acoustic Doppler Current Profilers (ADCPs) to investigate how turbidity currents drive hydro-sedimentary processes across different spatial and temporal scales. Specifically, we (i) characterise bedform morphologies within the channels, (ii) assess long-term channel evolution, and (iii) link observed changes to a recorded turbidity current. By quantifying how a discrete flow event can reshape the lakebed and drive channel evolution, this study provides insights into lacustrine turbidity current dynamics. Furthermore, we systematically compare observations from Lake Brienz with well-documented marine analogues, establishing whether lacustrine turbidity currents follow similar hydrodynamic principles or exhibit distinctive dynamics.

### Subaquatic channels dominated by cyclic-step morphology

High-resolution bathymetric mapping of the Aare Delta of Lake Brienz^37^ reveals a distinctive multi-channel network that evolves from three proximal branches (Northern (N), Main (M), and Southern (S) channels), reduces to two as N merges into M, and finally converges into a single downslope channel that reaches the basin floor (Fig. 1c). In unconfined areas of the delta slope, undulating bedforms with sinuous crest lines occur (Fig. 1c). In contrast, the bedforms confined within the channel system, particularly in Channels M and S, appear morphologically distinct, with crescentic, upward-convex geometries and shorter wavelengths^37^ (Fig. 1c). Oriented transversely to the flow direction, these features form a characteristic staircase-like morphology within the channels (Fig. 1e) and are widely interpreted as cyclic steps formed by turbidity currents^38^. Cyclic steps develop under non-uniform flow conditions, where acceleration over the steeper lee face induces erosion, while deceleration in the downstream trough favours deposition on the flatter stoss side^38–40^ (Fig. 1e). Thus, the bedform train migrates upslope over time with successive flows despite the overall downslope direction of sediment transport^38,41,42^.

**Fig. 2:**
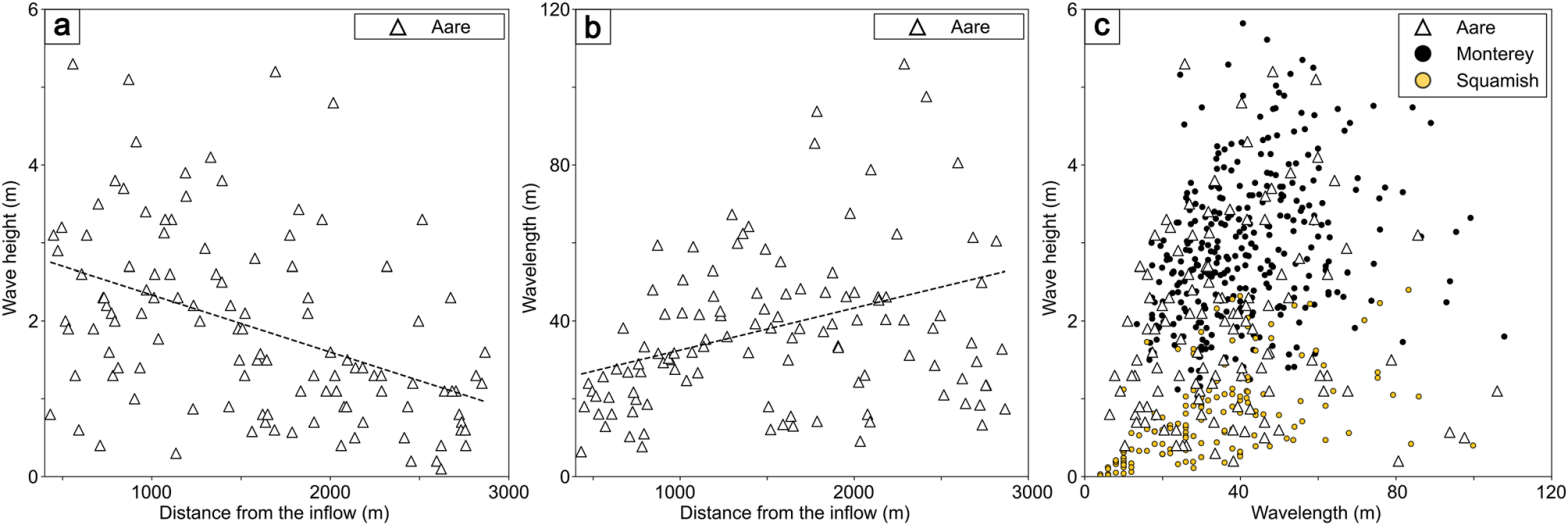
Scatter plots of (**a**) wave height and (**b**) wavelength of cyclic steps as a function of distance from the inflow, measured from dip-oriented profiles along the thalwegs of Channels M and S in the Aare Delta. Linear regressions highlight the down-channel trends. (**c**) Comparison of aspect ratio (wavelength/wave height) for the Aare Delta (white) with the Squamish prodelta (yellow) and Upper Monterey Canyon (black), modified after^43^.

The morphology of cyclic steps is influenced by multiple parameters, including slope, grain size, sediment concentration, triggering mechanism, flow velocity and thickness which together shape these bedforms^15,24,38,39,43^. All these factors vary along subaquatic channels and across environments, producing differences in wavelength, height and overall bedform shape^15^. Several studies have described these bedforms by their aspect ratio (wavelength/wave height), a metric that provides insights into the nature of the flow^15,38,43,44^. In the Aare system, morphometric parameters were extracted from profiles along the thalwegs of Channels M and S. In the upper reaches near the inflow, bedform wave heights ranged from 3 to 5 m (Figs. 1c, 2a) with wavelengths of 25–50 m (Figs. 1c, 2b). Further downstream, while wave heights decreased to 0.5–3 m (Figs. 1c, 2a), wavelengths increased to ~ 100 m (Figs. 1c, 2b). These downstream trends are highlighted by linear regressions in Figs. 2a and 2b.

Comparable scales, morphology, and down channel trends have been observed within subaquatic channels globally^44^ and align with predictions from numerical models^38,39^. Their occurrence has been documented both in marine turbidity-current systems^24,38,42,44–48^ and in lacustrine settings^13,15,17,49^. Geometric characteristics of cyclic steps such as wavelengths and heights can offer some constraints on flow behaviour^38^, for example, velocity, flow thickness, and sediment concentration^38,47^. However, the strong interdependence between flow parameters makes it difficult to isolate the role of any single factor in natural settings^15^*.*

Steeper delta fronts (e.g., Squamish prodelta, Canada^42^, Capbreton, France^50^) produce shorter‐wavelength, taller steps, whereas gentler slopes (e.g., Wabush Lake, USA^49,51^, Monterey upper canyon, USA^45^) generate longer wavelengths and lower amplitudes^15^. Well-defined channels tend to host more distinct crescentic cyclic steps; however, wavelengths systematically increase downslope as slope decreases^15^ as observed within the Aare system (Fig. 2b).

To place our observations in context, we compared the Aare Delta cyclic steps with those from two well-characterised settings: the Squamish prodelta and Monterey Canyon (Fig. 2c). The Squamish Delta, located in a fjord within Howe Sound, British Columbia, Canada, receives over 1 × 10⁶ m^3^/yr of sediment from the Squamish River. Its prodelta slope hosts three large submarine channels that terminate into depositional lobes 1–2 km from the delta lip^19,41–43,46,52^. Monterey Canyon is an extensive ~ 400 km-long submarine canyon–channel system in Monterey Bay, California, USA, which receives 0.2–0.5 × 10⁶ m^3^/yr of sediment from littoral drift from nearby rivers and coastal erosion^18,22,23,43,53^. Cyclic steps in the upper Monterey Canyon are more symmetrical and possess smaller aspect ratios (wavelength/wave height) compared to the Squamish prodelta^43^. These differences have been attributed to varying slope gradient, or specific flow properties^43^.

The cyclic step morphologies of the Aare channels fall within the range observed in both the Squamish prodelta and the upper Monterey Canyon. Proximal cyclic steps with higher wave heights and shorter wavelengths are comparable to Squamish, whereas downstream steps with longer wavelengths resemble those of Monterey (Fig. 2c). Despite receiving only ~ 0.05 × 10⁶ m^3^/yr of sediment and extending just a few kilometres, the Aare channels reproduce within a compact setting a continuum of morphologies that in larger systems is expressed over greater distances. This condensed expression of cyclic steps underscores the value of lakes as natural laboratories for investigating processes that are expressed at larger scales in marine environments.

### Channel dynamics and temporal evolution

**Fig. 3:**
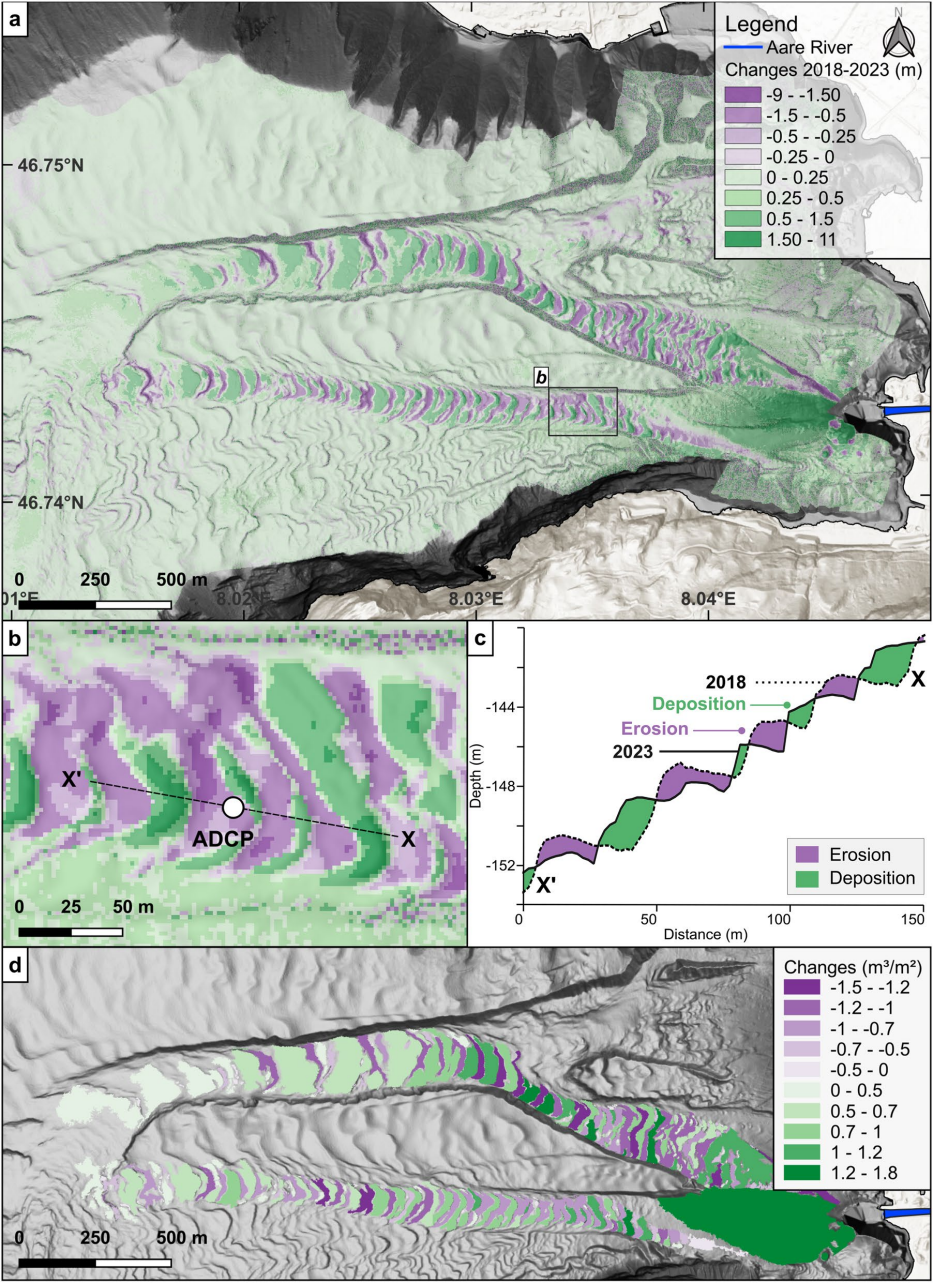
(**a**) Digital Bathymetric Model of Difference (DoD) between 2018 and 2023 (m). Erosion is shown in purple, and deposition in green. Changes are quantitatively represented using a non-linear 8-class legend, capturing the full range of both major and minor variations. The black rectangle outlines the extent shown in (**b**). (**b**) Enlarged view of Channel S at the ADCP location. Profile shown in (**c**). (**c**) Channel thalweg profile (E–W) illustrating cyclic step migration. (**d**) Volume of change per patch, normalised by area (m^3^/m^2^), showing a decreasing trend down-channel. Maps generated in QGIS 3.34 (https://qgis.org) and edited in Inkscape 1.4 (https://inkscape.org). Sources: Federal Office of Topography Swisstopo and Esri World Hillshade via QuickMapServices. CRS: EPSG:4326.

To further investigate the dynamics within the Aare channels, repetitive surveys have been conducted, which document how cyclic steps migrated over time*.* A differential bathymetric model, obtained by subtracting the 2018 from the 2023 surveys^16^ (Fig. 3a), reveals substantial lakebed change over the five-year period. Near the Aare inflow, deposition reached up to 11 m, while sediment accumulation across the broader delta region generally remained below 0.3 m. Channel N (Fig. 1c) experienced localised erosion but showed overall little change. In contrast, both Channels M and S (Fig. 1c) show alternating zones of erosion and deposition (Figs. 3a, b), reflecting downslope sediment transport and the associated upslope migration of cyclic steps driven by stoss-side erosion and lee-side deposition. In the downstream segments, cyclic steps clearly shifted upslope (Supplementary Fig. S1, Supplementary Vid. S1). In contrast, in the upper channel reaches, where wavelengths are shorter, tracking individual crests becomes ambiguous (Figs. 3b, c), suggesting migration may have surpassed a full wavelength (Supplementary Fig. S1, Supplementary Vid. S2).

Figure 3d displays the average volumetric change per unit area (m^3^/m^2^) within each migration pattern, showing that both erosion and deposition decrease with increasing distance from the inflow, a trend particularly evident in Channel M. This downstream spatial variation of changes reflects the cumulative imprint of turbidity currents over the five-year period. In the steep, narrow upstream sections, the presence of erosional to transportational cyclic steps^54^ suggests that flows stayed sediment-laden and self-sustaining^1,6^. In these areas, flows were able to generate larger-magnitude changes per surface area on the lake floor. Downslope decreasing slope angle and increasing channel width likely caused the turbidity currents to decelerate, lose erosive capacity, and become more dilute^54^. This resulted in a dominance of depositional cyclic steps^54^ and generally smaller magnitudes of change.

The evolution of turbidity currents observed in oceanic channels aligns with the down-channel erosion–deposition trends observed in the Aare system (Fig. 3d). Similar downstream behaviour has been documented in in the Congo Canyon, a large deep-water submarine system extending hundreds of km offshore West Africa^55^, where less frequent flows were detected in the lower reaches, suggesting that many flows lose energy and dissipate downstream^55^. Similarly in the Bute Inlet channel system, a 50 km long channel system in British Columbia, Canada^56,57^, turbidity current frequency decreased with increasing distance along the channel^57^ and of the 113 flows detected by the most proximal ADCP, only 2 reached the furthest point^57^. Transport to the terminal lobe was driven by infrequent, high-magnitude turbidity currents whereas most flows decelerated and deposited sediment in the upper channel, replenishing these proximal reaches^56^. Sediment in the proximal reaches can be reworked by later turbidity currents, sometimes multiple times, before reaching the basin^57^. These observations appear consistent with the sediment dynamics observed in the 2.5 km-long Aare channels, suggesting turbidity current activity is greater in the proximal reaches, reinforcing its applicability at smaller spatial scales in lacustrine systems.

### Turbidity current monitoring

ADCPs deployed during the summer of 2022 enabled direct observation of flow activity within Channels M and S of the Aare Delta (Fig. 1c), ultimately linking the geomorphologic changes to flow processes. In perialpine lakes, riverine inflows typically intrude into the pycnocline, particularly during summer months when a sharp, temperature-induced density gradient develops at depths of 20–30 m^58^, while deeper water remains largely quiescent aside from low-magnitude, long-period internal waves driven by wind^59^. Turbidity currents occur episodically when the inflowing water becomes dense enough, usually during high-sediment pulses^58^, overcoming lake stratification. These flows are detected by the ADCP as simultaneous increases in bottom velocities and sediment concentration^20^.

**Fig. 4:**
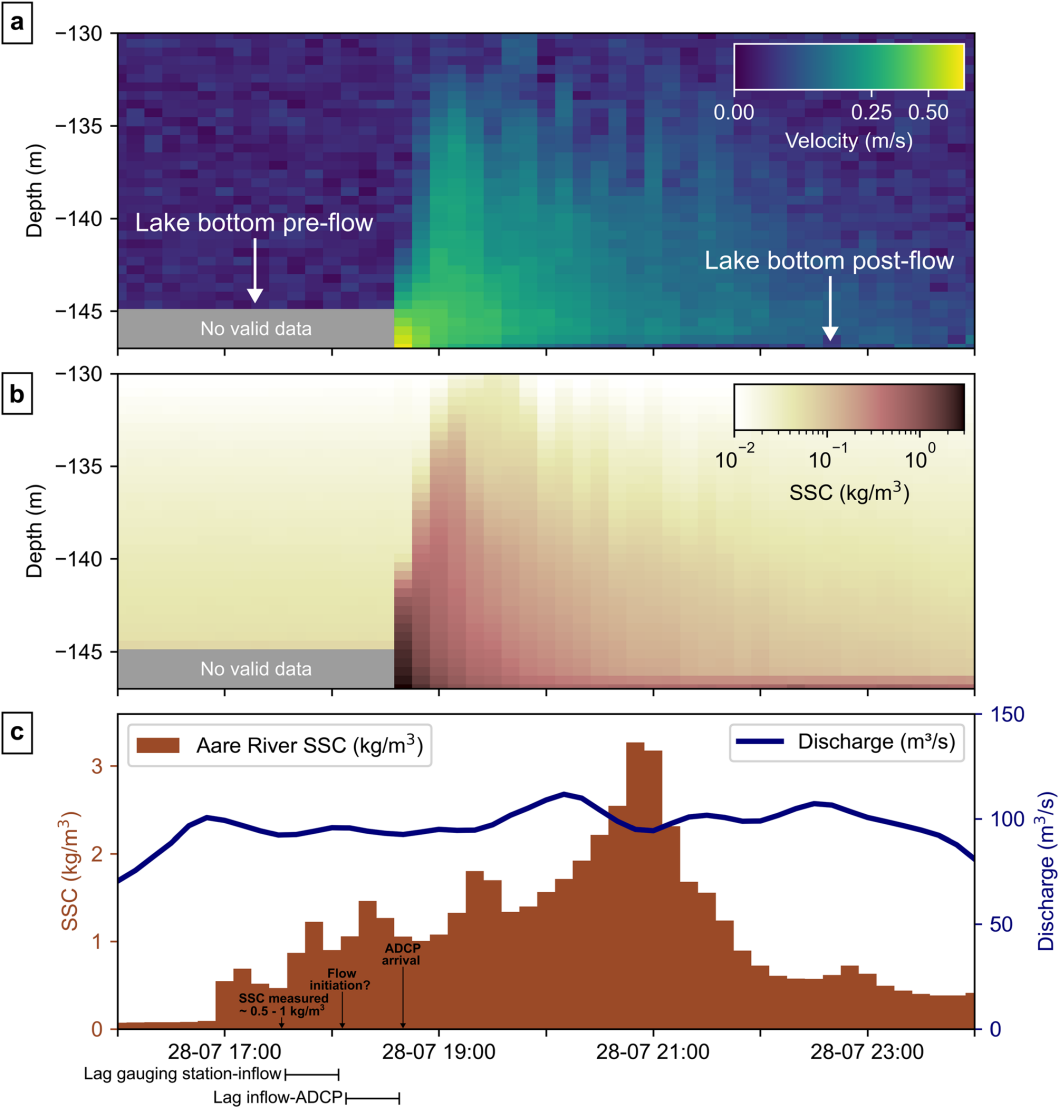
Turbidity current observed on July 28, 2022. (**a**) Water-column velocity (m/s). (**b**) Water-column Suspended Sediment Concentration (SSCchannel, kg/m3), obtained from inversion of the acoustic backscatter. Grey‐shaded areas in (**a**) and (**b**) mark regions below the detected lakebed where no valid ADCP measurements were recorded. Backscatter data and validity checks confirm that this level corresponds to the lakebed prior to the event. (**c**) Aare River discharge (m3/s) and SSCriver (kg/m3) measured 3.4 km upstream at the Aare-Brienzwiler gauging station. With estimated river velocities of ~ 2–3 m/s from the gauging station to the inflow and turbidity current velocities of ~ 0.6–1 m/s from the inflow to the ADCP, the resulting travel times imply a lag of approximately ~ 1 h between gauging station measurements and ADCP detection.

The turbidity current with the highest measured velocity during the deployment occurred on July 28 in Channel S (Fig. 4) and was first detected by the ADCP at 18:40 UTC, coinciding with a period of elevated but stable river discharge of 100 m^3^/s (Fig. 4c). River suspended sediment concentration (SSC_river_) increased from a baseline of 0.05 kg/m^3^ at 17:00 UTC to a peak of 3 kg/m^3^ at 21:00 UTC (Fig. 4c). Temperature-induced density differences in freshwater typically range from 0.13^27^ to 0.25 kg/m^3 12^, and rarely exceed 1 kg/m^3 27^. When accounting for the travel time from the gauging station to the ADCP (Fig. 4c), the measured SSC_river_ of 0.5—1 kg/m^3^ in the hour preceding flow initiation would have been sufficient to trigger hyperpycnal plunging (Fig. 4c). Yet, SSC_river_ continued to increase after the turbidity current. However, higher sediment concentrations did not sustain or trigger further flow activity (Fig. 4c). Additionally, several other periods with equal or greater SSC_river_ did not lead to flow initiation. These observations suggest that high sediment input from the inflow alone may be insufficient to trigger turbidity currents. Instead, the presence of mobile and erodible sediment at the delta front^41,58^ likely serves as a necessary precondition^19^.

Within the channel, a peak velocity of 0.65 m/s and maximum SSC_channel_ of ~ 2.5 kg/m^3^ were recorded in the deepest measurement bin, just above the lakebed, at the onset of the event (18:40 UTC; Fig. 4a). However, these derived SSC_channel_ carry substantial uncertainty, as the assumed grain size (60 µm) used for the backscatter inversion is based on sedimentological characteristics of the channel floor^34^ and cannot be constrained within the turbidity current. Different choices in grain sizes affect the estimated concentration and therefore flow density considerably (Supplementary Fig. S2)^1,60,61^.

The current maintained a consistent east-to-west direction (270° ± 20°) for 5 h, aligned with the channel axis, and began to lose directional coherence after 23:40 UTC. Flow thickness increased during the first 80 min, reaching up to 15 m before gradually decreasing alongside channel velocities and SSC. After 10 h, conditions had returned to background lake velocities. In the considered event, deeper acoustic returns were recorded from the onset of the event and persisted throughout the deployment (Fig. 4a). The thinner frontal cell, sustained by continuous channel-floor erosion^1,56^, suggests that the observed deepening reflects local erosion.

During summer 2022, the ADCP deployment revealed about one flow per month in the Aare system, with observed peak velocities of 0.45 m/s in Channel M (July 4) and 0.65 m/s in Channel S (July 28). This is lower than in Bute Inlet (~ 5 per month, up to 6.2 m/s^57^), the Squamish Delta (> 25 per month, up to 3 m/s^42,47^), or Monterey Canyon (~ 1 per month, up to 7.2 m/s^18^), indicating that both the frequency and magnitude of turbidity currents in the Aare Delta are lower than in oceanic systems. These differences may reflect the limited monitoring window of this study, reduced sediment supply, or the inherent scales of lacustrine systems with shorter runout distances and shallower depths.

### ADCP circular dynamic bathymetric mapping

Although the maximum velocity recorded (0.65 m/s) is modest compared to flows exceeding several m/s in deep-sea canyons^20,42^, it appears to have been sufficient to induce channel reworking beneath the ADCP, as indicated by our data (Fig. 4a). The following section analyses how this event contributed to the multi-year morphological changes identified in repeated multibeam bathymetric surveys. While ADCPs are primarily designed for current measurements, their along-beam bottom detection can be used to reconstruct small-scale bathymetry of the channel floor below the mooring^24,42^. During the deployment, the ADCP rotated slowly around its axis as the supporting rope twisted back and forth under environmental forcing, causing its four beams to sweep across a circular footprint beneath the instrument. Echo intensity from each beam provided four bottom detections per measurement, with compass readings used to determine their respective orientations. Over several weeks, this approach enabled reconstruction of the lakebed along an 18-m circular perimeter, allowing for comparative pre- and post-turbidity current analysis of the channel floor. Since data were only collected along the perimeter, the inner surface was interpolated based on peripheral depth values and guided by typical geometries observed at the site (Fig. 3b).

**Fig. 5:**
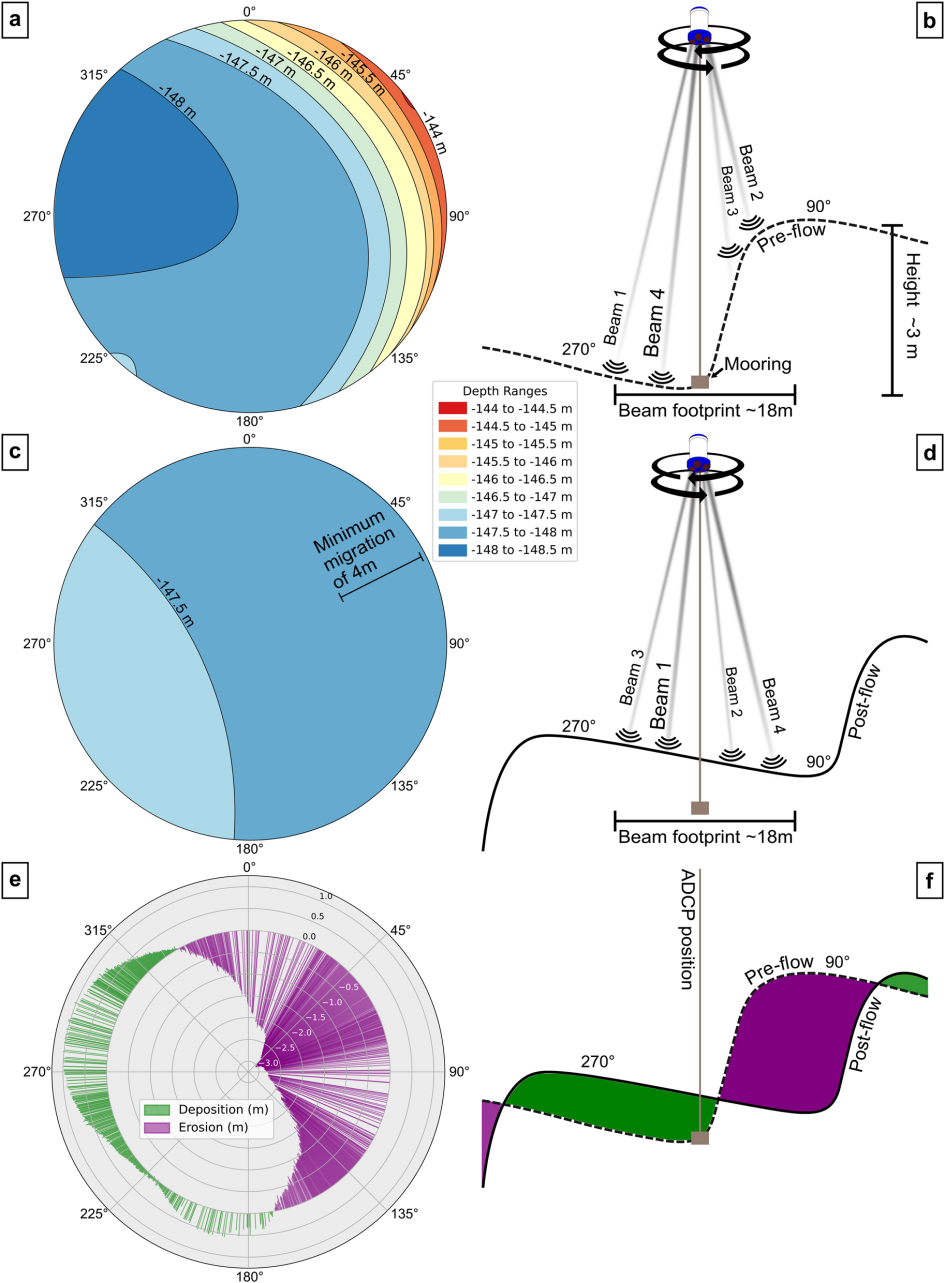
(**a**) Pre-flow depth measurements (27,032 individual points) within an 18-m diameter area covered by the ADCP footprint, determined by the instrument’s height above the bottom and the beam angle. Depth is shown as a function of heading, based on perimeter measurements with interpolation within the footprint. (**b**) Pre-flow morphology interpretation sketch between 90° and 270°. (**c**) Post-flow depth measurements (26,800 individual points) and interpolation. (**d**) Post-flow morphology interpretation sketch between 90° and 270°. (**e**) Depth differences calculated as post-flow minus pre-flow (c minus a). (**f**) Interpretation sketch illustrating the observed bathymetric changes.

ADCP bathymetric mapping in the month prior to the July 28 event (Fig. 5a) revealed an elevation difference of ~ 3 m along the circular perimeter covered by the instrument’s beams, ranging from –144.5 m at 350° to –148 m at 270°, matching the orientation and height of nearby cyclic steps (Fig. 3b). This suggests that the ADCP detected both a stoss and a lee face (Fig. 1e), as illustrated in the schematic profile (Fig. 5b). Pressure remained stable before and after the event, confirming that the mooring itself was not displaced down-channel (Supplementary Fig. S3). Depth measurements acquired after the event (Fig. 5c) showed nearly uniform values of –147.5 m across the footprint, indicating that the bedform had migrated beyond the instrument’s range, leaving the beams directed at a single stoss face and indicating up to 3 m of vertical erosion (Fig. 5d). Such migration explains why velocity measurements appeared shallower before the flow (grey areas, Fig. 4a), as two out of the four beams were initially systematically obstructed by the higher stoss, preventing measurements below -144 m. After the bedform migrated, all beams reached -147 m, allowing for deeper readings (Fig. 4a).

During the first 10-min interval following the flow’s arrival, the ADCP already indicated measurements until a depth of –147 m, indicating that most of the bedform migration occurred rapidly at the onset of the turbidity current, within a period shorter than the instrument’s resolution. Although uncertainty remains about whether the cyclic step crest lay at the edge or centre of the footprint, the lee faces measured at the site are ~ 4 m long (Fig. 3b), providing a conservative minimum estimate of upslope migration. The July 28, 2022, turbidity current (Fig. 4) therefore contributed to some of the 20–35 m upslope migration of cyclic step characterised between 2018 and 2023 in repeated multibeam surveys at the ADCP location (Fig. 3b). Similar migration magnitudes and rates have been shown in the Squamish prodelta^41,42^. Hage et al.^41^ reported 1.5 m of upslope displacement over 12 min associated with velocities greater than 2 m/s, while Hughes Clarke^42^ observed 5–7 m of upslope migration as the net result of seven flows (0.5–3 m/s) over a 2-h period. However, only two flows displaying a denser near-bed basal layer^18^, appeared to cause migration. In one case, an individual flow with velocities of 2.5–3 m/s initiated rapid upslope displacement, marked by lee-face erosion during the first 20 s, and sustained for a further 90 s with accompanying stoss-side accretion^42^. A comparable migration magnitude of at least 4 m (Fig. 5) was documented in this study. Although high-density, near-bottom velocities were not directly observed due to instrumental limitations^42,53^, we hypothesise that such a layer may have been present and responsible for the migration. Additionally, migration rates remained unresolved because of the limited sampling rate of our setup, and may have been as fast as those reported in the Squamish prodelta^42^.

## Conclusion

Monitoring turbidity current dynamics in lacustrine settings remains scarce compared to marine environments, thereby limiting our ability (i) to evaluate how sediments are transported in lakes and (ii) to use lakes as field-scale laboratories for oceanic systems. This study explored a turbidity current in the Aare Delta of Lake Brienz (Switzerland). We provide direct multi-temporal and multi-scale observations linking a single turbidity current to long-term channel evolution. At the scales of minutes and metres, an individual cyclic step migrated in response to a single flow event. Over multi-year intervals, the cumulative effect of flows explains the reorganisation of bedforms within the channels, as captured in repeated bathymetric surveys. Comparisons with well-documented marine analogues highlight overall similarities in cyclic step morphologies, migration patterns, and general flow behaviour, although the systems differ in their broader characteristics. These observations support the view that lakes serve as valuable scaled-down analogues for studying the dynamics of larger marine systems. Conversely, insights from marine environments provide a framework for understanding lacustrine turbidity currents.

Some challenges in understanding the Aare Delta system remain. The 3-month observation window leaves it uncertain whether the deployment period captured representative flow magnitudes and frequencies. Key processes such as flow partitioning between the two channels, their subsequent evolution toward the basin, and their role in shaping the channel system and associated deposits remain poorly constrained. Triggering mechanisms are likewise unresolved: although flows coincided with periods of elevated river discharge and suspended sediment, their frequency was lower than expected given the number of hyperpycnal conditions observed during the deployment. Additionally, other types of flow triggers (e.g., flood-driven or slope-failure events), as well as the potential diversity in their internal structure, have yet to be documented and addressed by future studies.

Lacustrine systems offer valuable monitoring opportunities. Apparent lower event frequencies and magnitudes may hinder observations, underscoring the global need for longer-term monitoring to capture a wider range of events^4,14^ and improve our understanding of turbidity currents in freshwater globally. At the same time, their reduced size, typically a few km, provides an intermediate scale between vast oceanic systems and m-scale flume experiments which are often constrained by scaling uncertainties^18^. From this perspective, lacustrine settings also offer ideal test sites for new approaches. In particular, recent advances in passive and system-wide monitoring using distributed hydrophones, geophones and fibre optic sensing^62–64^ now open possibilities for capturing turbidity current initiation and overall evolution. This study highlights the potential of lakes as accessible, cost-effective natural laboratories for advancing our understanding of turbidity currents across all temporal and spatial scales.

## Methods

### Setting

Lake Brienz is a Swiss perialpine lake reaching a maximum depth of 260 m, covering 29.8 km^2^, and holding a volume of 5.1 km^3,65^. Sedimentation is almost entirely clastic, controlled mainly by the inflows of the Aare and Lütschine Rivers, which enter at the eastern and western ends, respectively, forming substantial deltas^33,65^. Sedimentation rates are high compared to other Swiss lakes, with accumulation reaching 3 cm/yr in the central basin and up to 4.7 cm/yr near the deltas^37^. The Aare River drains a catchment of 555 km^2^, of which 16% is glaciated. Its average annual discharge is 35 m^3^/s, with a pronounced seasonal pattern: ~ 63 m^3^/s during summer (June–August) due to snowmelt, and ~ 17 m^3^/s in winter (December–February). A major flood in August 2005 produced a peak discharge of 444 m^3^/s. The river delivers ~ 0.13 × 10^6^ t/yr of suspended sediment^66^, which corresponds to ~ 0.05 × 10^6^ m^3^/yr assuming bulk density of 2650 kg/m^3,37^.

The Aare Delta lies within a steep bedrock-bounded basin, a setting where underflows originating from the inflow influence deltaic morphology, progradation rates, and depositional transitions, as shown in theoretical and experimental studies of hyperpycnal deltas^67–69^. However, human activities have markedly altered the natural trajectory of delta evolution. Between the 1930s and early 1950s, several hydroelectric dams were constructed, reducing sediment supply to Lake Brienz by a factor of 2.8 compared to pre-dam conditions^66,70^. Since the 1940s, gravel extraction from the subaquatic delta has further decreased sediment input. At the end of the nineteenth century, the Aare was rerouted and confined to a single outlet. As the river has only been able to discharge its sediment at a single point since the rerouting, focused accumulation has resulted in recurrent deltaic collapses^35,36^ described as human‐induced autocyclic failures^36^. Together, these factors (e.g., damming, gravel extraction, and deltaic failures) limit modern deltaic progradation.

The subaerial rerouting of the inflow also impacted the sub-lacustrine channel system with new incisions^34^, giving rise to the present southern channel (S) and possibly the upper reaches of the main channel (M). Bathymetric mapping (Fig. 1c) reveals a branched network of three principal channels: northern (N), main (M), and southern (S). These merge into a single channel ~ 3 km downstream of the inflow (Fig. 1c).

### Aare River gauging station

Aare River discharge (m^3^/s), turbidity (BSTU), and temperature (°C) are monitored at the automatic Aare-Brienzwiler gauging station, located 3.4 km upstream from the river mouth, at 10-min intervals. Suspended sediment concentration (SSC; kg/m^3^, Fig. 4) was obtained through a linear regression with SSC values from water samples collected two to three times per week at the gauging station and analysed in the laboratory, providing a continuous 10-min SSC time series (Supplementary Fig. S4).

### High-resolution repetitive bathymetric surveys

Bathymetric mapping of the Aare Delta was carried out in May 2018 and November 2023 using a Kongsberg EM2040 multibeam echo sounder (Kongsberg Maritime, Horten, Norway) and processed with CARIS HIPS/SIPS software version 10.4 (Caris, Fredericton, Canada), following identical methodologies for both surveys. Detailed survey and processing information can be found in Fabbri et al.^37^. Surfaces were generated with a grid size of 2 × 2 m. Geoprocessing tasks were performed in QGIS version 3.36.1, including cyclic step morphology picking (used in Fig. 2), difference maps (Fig. 3), and volume calculations (Fig. 3d). To better visualise down-channel change trends, we quantified volumetric change for each delineated polygon (Fig. 3d). For each polygon, vertical changes were integrated across all grid cells, summed to obtain the total change, and normalised by area. This approach highlights trends that are not readily apparent in the pixel-wise difference map (Fig. 3), due to the overall symbology and the non-uniformity of changes within individual bedforms.

### Acoustic Doppler Current Profilers (ADCPs)

Data were collected during a three-month deployment from June 10 to September 14, 2022, using 600-kHz downward-looking Teledyne Workhorse Sentinel ADCPs (Teledyne Marine, Poway, CA, USA). Two ADCPs were moored 1 km downstream of the inflow along the channel’s thalwegs in Channels M and S, respectively. Here we focus on data from Channel S (Figs. 1c-e). The instrument was located at a depth of 120 m, positioned 27 m above the lake floor (Fig. 1d). Measurements were acquired at 0.5 m vertical bins every 2.4 s and averaged into 10-min ensembles, comprising 250 pings per ensemble.

Suspended sediment concentrations were quantitatively derived from the acoustic inversion of the backscatter using a representative grain size of 60 µm, consistent with the mixed silt–sand (~ 63 µm) composition observed on the channel floor^71^. The inversion was implemented in Python with the Hydrac package^60,72^.

The four ADCP beams, oriented at 90° to each other, also functioned as individual echosounders for bathymetric mapping^24^. To ensure valid comparisons, we first verified from the pressure sensor data that the instrument experienced no down‐channel displacement during the deployment (Supplementary Fig. S3). Bottom reflections were acquired over two extended periods (> 40 days, before and after the flow), taking advantage of the ADCP’s slow rotation, with compass readings used to locate individual soundings. This rotation, determined at 0.1°, enabled circular bathymetric mapping along the periphery covered by the beams. Figure 5e shows the headings at which values could be acquired both before and after the flow. Depth values collected while the instrument rotated rapidly, such as during the flow, were excluded to avoid artefacts. Processing was performed in Python, and plots were generated using Matplotlib.

## Supplementary Information

Supplementary Information 1.

Supplementary Information 2.

Supplementary Information 3.

Supplementary Information 4.

Supplementary Information 5.

## Data Availability

River parameters were sourced from the Aare-Brienzwiler gauging station [https://www.hydrodaten.admin.ch/en/seen-und-fluesse/stations/2019] , operated by the Federal Office of Environment (FOEN), with data available at 10-min resolution [https://www.bafu.admin.ch/bafu/en/home/topics/water/data-and-maps/water-monitoring-data/hydrological-data-service-for-watercourses-and-lakes.html] Single survey bathymetric maps for Swiss lakes (e.g., Lake Brienz from 2018) can be visualised on swissBATHY3D [https://map.geo.admin.ch/] and downloaded from the Federal Office of Topography – Swisstopo [https://www.swisstopo.admin.ch/en/height-model-swissbathy3d]. All data used in this study are provided in the Supplementary Information under the Source_Data folder.
