# Supplementary material for: Direct observations of bedform migration driven by turbidity currents in a lacustrine channel
Source: Sci Rep. 2025 Oct 30;15:38026. doi: 10.1038/s41598-025-21833-6 (PMC12575640; doi:10.1038/s41598-025-21833-6)
Supplement: Supplementary file 2 — Supplementary Information 2. [file 41598_2025_21833_MOESM2_ESM.docx]

# Figure S1: Overview of the two areas covered by Videos S1 and S2

Cyclic step migration observed in the channels based on bathymetric surveys from 2018 and 2023 is illustrated in Videos S1 and S2, provided in GIF format. (Graphical Interchange Format) accompanying this document. An overview of the channel areas corresponding to the two videos is shown in Fig. S1.

Figure S1: Overview of the two areas covered by Video S1 and Video S2. Map generated in QGIS 3.34 (https://qgis.org) and edited in Inkscape 1.4 (https://inkscape.org). Sources: Federal Office of Topography Swisstopo and Esri World Hillshade via QuickMapServices. CRS: EPSG:4326.

# Video S1: Cyclic step migration (2018-2023) in the lower reaches of Channel N

Video S1 shows a location in the lower northern channel where crest tracking is straightforward, making aliasing unlikely and upslope migration is clearly visible and quantifiable.

# Video S2: Cyclic step migration (2018-2023) in the upper reaches of Channel S

Video S2 shows the situation near the ADCP, where crest tracking proves more challenging because (i) the wavelength is much shorter and (ii) migration is more significant. Consequently, quantifying migration at this location is not possible, as it possibly exceeded a full wavelength.

# Figure S2: ADCP backscatter hydroacoustic inversion to estimate Suspended Sediment Concentration (SSC)

We used the inversion software Hydrac (<https://bitbucket.org/fromantgu/hydrac/src/master/>.), developed by Guillaume Fromant. The following parameters were selected for the inversion (presented in Fig.4b in the main text):

*## Temperature taken from the thermistors*

**No water parameters data… Please enter a mean temperature value to estimate sound attenuation:** 5.7

**Deployment mode:** Mooring

**Select a temporal window (in sec) for averaging of moored acoustic parameter instrument (0 for no averaging):** 0

**Select a vertical bin size (in m) for averaging of casted physical parameter instrument (0 for no averaging):** 0

*## For single frequency ADCP*

**Please enter a method amongst the following list [‘Implicit’]:** Implicit

**Please enter a distribution width:** 0.5

*##Grainsize radius in meters (e.g., a 60-micron diameter corresponds to 0.00003 m)*

Please enter a median radius: 0.00003

To assess the sensitivity of the computation inversions were performed using different grain sizes and distribution widths. A comparison of vertical SSC profiles at the arrival of the turbidity current (18:40 UTC, July 28, 2022) reveals substantial variability depending on the selected parameters. Figure S2 shows that, in our case, smaller grain sizes and narrower distribution widths yield significantly higher SSC values, highlighting the uncertainty associated with the estimates from Fig. 4b.


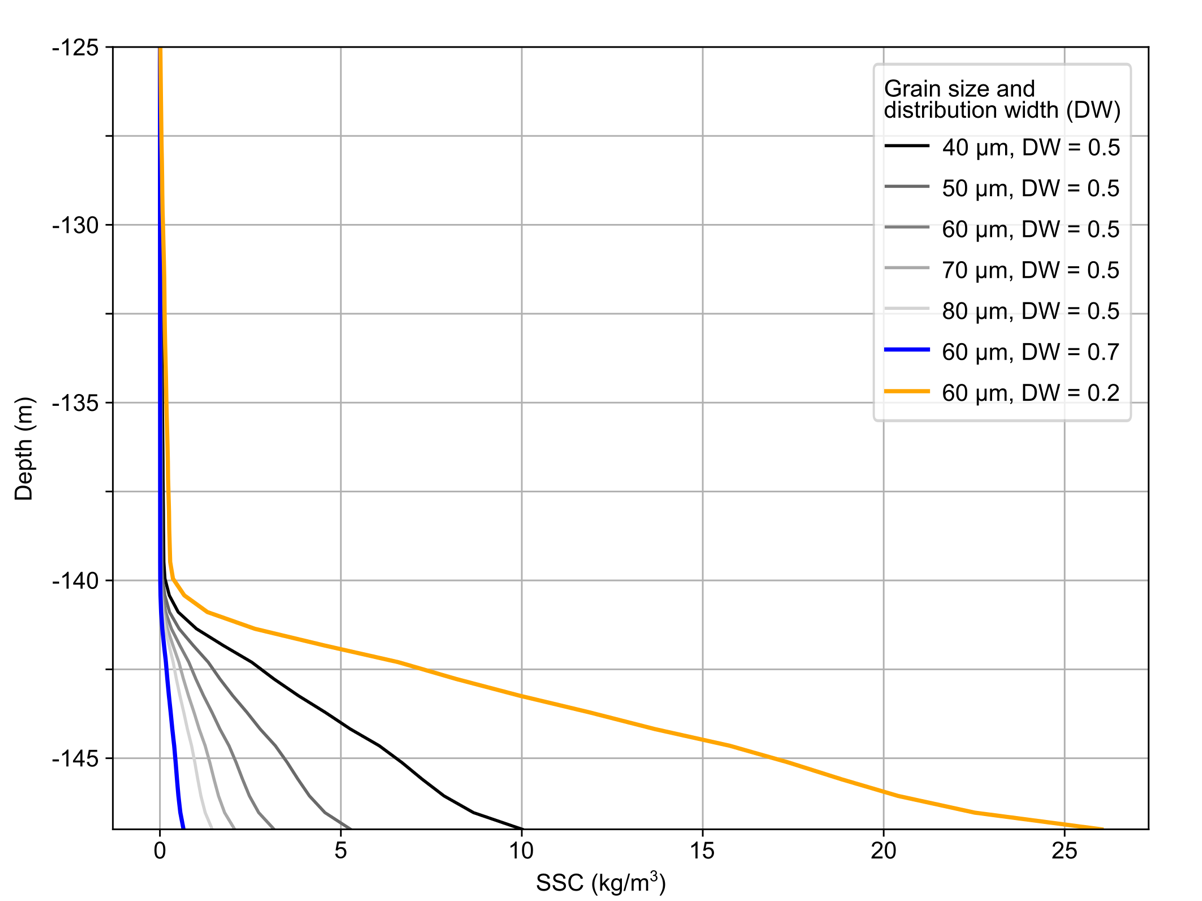


Figure S2: Suspended Sediment Concentration (SSC, kg/m³) profiles obtained using different grain sizes and distribution widths in the Hydrac software.

# Figure S3: Verification of the pressure sensor to assess any potential down channel displacement of the mooring

A combined temperature and pressure sensor (RBR Duet thermistor) configured to measure every 10 seconds, was positioned between the ADCP and the buoy at a depth of 70 m, recorded an identical mean pressure (Fig. S3) of 70.36 dbar before and after the flow. This consistency confirms that the mooring was not displaced downstream by the turbidity current, ensuring the ADCP's circular mapping remained applicable to the same area. Typically, an increase in pressure is observed during such events, caused by the instrument being pushed laterally and subsequently moving downward due to its attachment to the mooring. However, in this case, no such displacement was detected. This is likely because the turbidity current, affecting only the first few meters above the bottom, did not impact the instrument frame or the thin mooring line.

Figure S3: 24-hour record of pressure (dbar) data from July 28, 2022, as recorded by the RBR Duet thermistor placed along the mooring line. The black dot marks the arrival time of the turbidity current (18:40 UTC). Horizontal lines indicate the mean pressure before and after the event.

# Figure S4: Estimation of Suspended Sediment Concentration (SSC) in the Aare River using continuous turbidity data (BSTU)

Sediment transport in rivers is monitored in several watercourses, including the Aare River, by the Swiss Federal Office for the Environment (FOEN): <https://www.bafu.admin.ch/bafu/en/home/topics/water/state/water--monitoring-networks/monitoring-networks-for-sediment-transport-in-bodies-of-water.html>.

Since 1964, Suspended Solid Concentration (SSC) samples have been collected weekly or biweekly at the Aare-Brienzwiler gauging station for laboratory analysis (mg/l). Starting from July 4, 2017, a TS-Solitax probe (Hach) has been used to record turbidity optically at 10-minute intervals. The instrument measures light scattering at two specific angles: 90° for NTU (Nephelometric Turbidity Units) and 140° for BSTU (Backscatter Turbidity Units).

Episodic SSC measurements recorded between 2018 and 2023 were matched with their closest BSTU values. High concentrations tend to be under-represented. Outliers were removed from the dataset. Discrepancies may have arisen from probe saturation at high turbidity levels or from sampling bias, such as when operators collected samples near the riverbank, resulting in unrepresentative values. Additionally, the relationship between BSTU and SSC is known to depend on sediment grain size, and variability in grain size could explain the observed scatter. A linear regression was performed (Fig. S4) on 458 values (R² = 0.88), yielding the following equation:

SSC = 1.12 * BSTU -12.65 (equation 1)

To achieve a continuous estimation of SSC during the ADCP deployment, equation 1 was applied to BSTU measurements to derive SSC values (kg/m^3^). Negative SSC values predicted by the regression (corresponding to BSTU values below ∼10) were set to zero to avoid non-physical negative concentrations. This adjustment preserves the overall fit of the model while avoiding distortion of the relationship at higher concentrations.

Figure S4: Linear regression model using BSTU values to estimate Suspended Sediment Concentration (SSC in kg/m^3^). The analysis is based on 458 measurements collected between 2018 and 2023 after filtering outliers. The regression line (black) represents the relationship between BSTU (x-axis) and SSC (y-axis), described by the equation SSC=1.12⋅BSTU−12.65. The R^2^ value of 0.88 indicates the goodness of fit. The axes limits are fixed to 0–200 for clearer visualization.
