# Supplementary figures and images for: Direct observations of bedform migration driven by turbidity currents in a lacustrine channel
Source: Sci Rep. 2025 Oct 30;15:38026. doi: 10.1038/s41598-025-21833-6 (PMC12575640; doi:10.1038/s41598-025-21833-6)

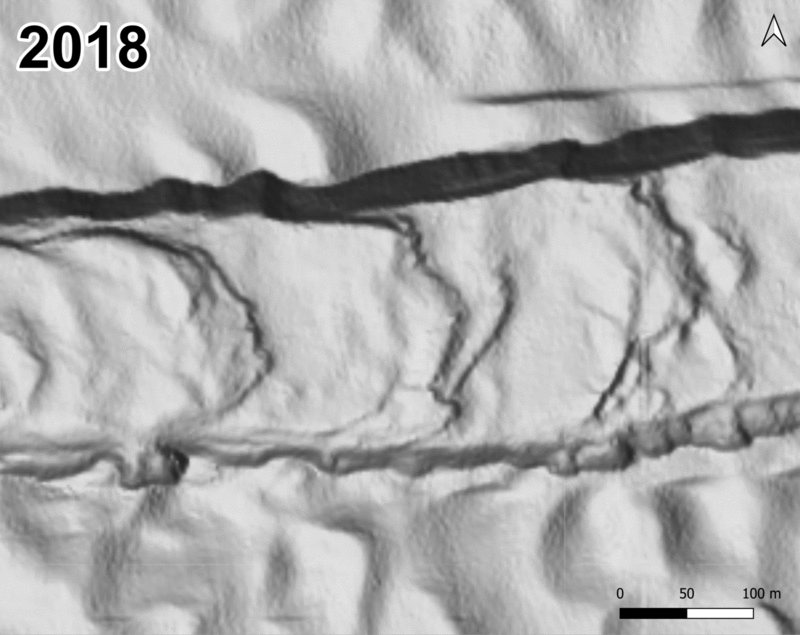

Supplement: Supplementary file 4 — Supplementary Information 4. [file 41598_2025_21833_MOESM4_ESM.gif]

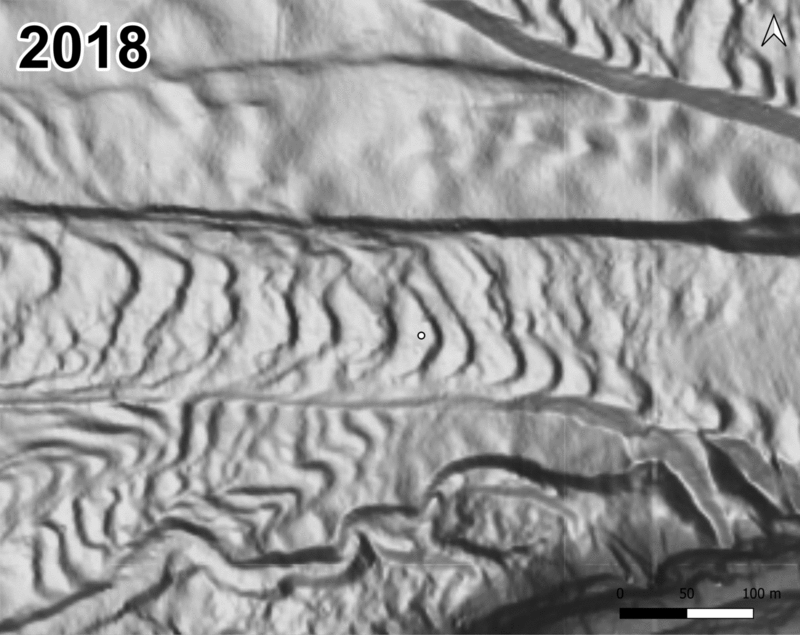

Supplement: Supplementary file 5 — Supplementary Information 5. [file 41598_2025_21833_MOESM5_ESM.gif]
